# Perceived Impeding Factors for Return-to-Work after Long-Term Sickness Absence Due to Major Depressive Disorder: A Concept Mapping Approach

**DOI:** 10.1371/journal.pone.0085038

**Published:** 2014-01-15

**Authors:** Gabe de Vries, Hiske L. Hees, Maarten W. J. Koeter, Suzanne E. Lagerveld, Aart H. Schene

**Affiliations:** 1 Program for Mood Disorders, Department of Psychiatry, Academic Medical Centre, University of Amsterdam, Amsterdam, The Netherlands; 2 Program for Mood Disorders, Pro Persona, Arnhem, The Netherlands; 3 TNO Work & Employment, Hoofddorp, The Netherlands; University of Electronic Science and Technology of China, China

## Abstract

**Objective:**

The purpose of the present study was to explore various stakeholder perspectives regarding factors that impede return-to-work (RTW) after long-term sickness absence related to major depressive disorder (MDD).

**Methods:**

Concept mapping was used to explore employees', supervisors' and occupational physicians' perspectives on these impeding factors.

**Results:**

Nine perceived themes, grouped in three meta-clusters were found that might impede RTW: Person, (personality / coping problems, symptoms of depression and comorbid (health) problems, employee feels misunderstood, and resuming work too soon), Work (troublesome work situation, too little support at work, and too little guidance at work) and Healthcare (insufficient mental healthcare and insufficient care from occupational physician). All stakeholders regarded personality/coping problems and symptoms of depression as the most important impeding theme. In addition, supervisors emphasized the importance of mental healthcare underestimating the importance of the work environment, while occupational physicians stressed the importance of the lack of safety and support in the work environment.

**Conclusions:**

In addition to the reduction of symptoms, more attention is needed on coping with depressive symptoms and personality problems in the work environment support in the work environment and for RTW in mental healthcare, to prevent long term sickness absence.

## Introduction

Major Depressive disorder (MDD) is a major cause of long-term sickness absence (LTSA) [1]–[6], and permanent work disability [7]–[10]. In addition, MDD contributes to prolonged sick leave duration in physical conditions such as low back pain and heart disease [11], [12]. MDD not only has adverse consequences for the individual employee, but also for their employer and society, due to costs related to loss of productivity, sickness absence, disability benefits and higher unemployment rates [13], [14].

Despite increasing efforts to help sick-listed employees with MDD to return-to-work (RTW), about 25% to 30% are still absent from work after one year [3], [15]. One explanation of this prolonged sick leave duration may be the course of MDD over time. Of the patients diagnosed with MDD about 21-37% will have a recurrent course within the first year while another 20% will not recover from MDD within two years [16], [17] and are diagnosed as chronic depression.

The duration of sickness absence related to MDD is predicted by several disease characteristics, such as an early age of onset, the duration and severity of MDD, and co-morbidity (i.e. anxiety, physical complaints and substance abuse) [18]–[20]. However, findings indicate that these disease characteristics alone are not sufficient for explaining the negative RTW outcomes [21]. A recent study showed that, although MDD symptom severity was one of the main predictors of disability, it could only explain 10% of the variance in disability outcome [20]. In addition, 50% of employees diagnosed with MDD through self-report were able to continue working, despite their symptoms [21], [22]. Furthermore, findings suggest that symptom recovery will not directly translate to improved RTW outcomes. This is illustrated by a Cochrane review, showing that regular mental health care only has limited effects on RTW, while these interventions are effective in reducing depressive symptoms [23]. In our own study aiming to identify factors that predict long-term RTW in sick-listed employees with MDD, we found that in addition to health factors (i.e. MDD severity and co-morbid anxiety disorder, work (i.e., work motivation) and personal (conscientiousness) factors were also predictive of long-term RTW [18]. Therefore, in sick-listed employees with MDD, multiple factors may play a role in explaining LTSA.

Previous studies have conceptualized work disability as the outcome of interactions between health, personal- and environmental conditions [24], [25]. Regarding personal factors, studies in common mental disorders (CMD's), such as anxiety disorders, somatoform disorders, and mild depression, have shown that a low level of education, a history of sickness absence, low self-esteem, low social functioning, older age (>50 years), and negative expectation regarding RTW all play a role in the duration of sickness absence [5], [19], [26], although findings are not always consistent [18], [19], [27]. In addition environmental factors, such as high job stressors [26], level of social support from colleagues and supervisor [5], and the possibility of accommodations at work [28] have been shown to influence the duration of sickness absence in CMD's. Surprisingly, personal and environmental factors that influence the duration of sickness absence specifically for MDD have hardly been studied [19].

In order to gain more insight in modifiable personal and environmental factors [19], [29], that impede RTW in employees with LTSA related to MDD, we examined the perspectives of employees, supervisors, and occupational physicians regarding these factors. Multiple perspectives were include because earlier work showed that stakeholders may vary in their views as to what they regard as important [30], [31]. For this study we used a combination of qualitative and quantitative research methods, a mixed-method design, which may be more suitable to capture the dynamic and complex nature of the RTW process [28]. The findings of the present study may help to identify employees with MDD who are at risk for LTSA in an earlier stage, and may improve professional support by the development and tailoring of RTW interventions.

## Methods

### Study design

Stakeholder perspectives on impeding factors for RTW were identified by concept mapping [32], a structured conceptualisation method, designed to organise and represent ideas regarding a specific theme. In addition, this method allows for the identification of similarities and differences between various stakeholder perspectives. Concept mapping combines qualitative individual and group processes with multivariate statistical analyses to help a group of individuals describe their views on a topic of interest and represent these views visually. Concept mapping has proven to generate valid and reliable results [33]. The combination of both qualitative and quantitative analyses makes it more data-driven than other qualitative research methods [33].

We submitted this research to the medical ethical committee of the Academic Medical Centre (Medisch Ethische Toetsingscommissie; MEC 06/258# 10.17.0923, date 18 june 2010). They declared that this research did not need an approval of this committee, because a medical intervention was not part of research. As no written informed consent was required, each participant was asked if he/she wanted to participate in this study.

### Participants

Participants were purposively sampled from the three key stakeholder groups who are directly involved in the RTW process: employees, supervisors and occupational physicians (OP's) [34]. To meet the inclusion criteria for this study, employees had to: a) be diagnosed by a psychiatrist with a Major Depressive Disorder (MDD) according to DSM-IV criteria, b) have a paid job, and c) have been on 100% sick leave for at least one year. The one-year criterion was selected in order to select employees who were unable to RTW, even though they received mental healthcare for a substantial period of time. In addition, duration of absenteeism is negatively related to the probability of a successful RTW [3], [35]. Employees were recruited through two large mental health care centres in the Amsterdam area. All employees we contacted participated in the study. Supervisors and OP's were included if they had directly supervised employees who did not RTW after sick leave due to MDD within one year. They were identified through their contact with the employees selected in our study (25% of the participating supervisors and 36% of the participating OP's). In addition, we recruited supervisors by contacting four companies (an elementary school, a high school, a prison and two healthcare institutions), who all selected one supervisor. Finally, OP's were also recruited by contacting two healthcare services with a response rate of 10% and 50%. Main reasons for non-response were due to either a lack of experience with this specific patient population and/or a lack of time to participate in this study.

### Data collection

The concept mapping procedure comprises of five steps:

1. Focus question and sampling of participants; Concept mapping starts with a single-focus question, described in the present study as: *“Which factors (work, personal and/or other factors) have contributed to the fact that you have (or your employee/patient has) not been able to return-to-work within one year of being on sick leave*?” For employees, the focal question pertained to their own experience, for supervisors and OP's, the focal question referred to their experience as a professional. Professionals could refer to more than one case, as they usually had had experiences with several employees in their caseload who did not return-to-work within one year.
2. Generation of statements; Next, participants were asked to generate statements pertaining to the focal question, based on their own experiences. All statements were written down by the researcher as they were expressed by participants. Two groups of researchers, two researchers each, then independently eliminated (a) statements that were unclear or unrelated to the focal question, and (b) redundant statements. In a consensus meeting, both groups of researchers presented the results of the cleaning phase to each other. In case of differences, a consensus decision was made. Reduction of statements was done in order to control for the complexity of the following steps [36].
3. Prioritization and categorization; Prioritization and categorization of the final set of statements was done individually by each participant. Prioritization implies that respondents prioritize statements by dividing them into five groups of equal size. Group one was defined as least important impediment on RTW and group five as most important impediment on RTW. Categorization means that participants were asked to put together those statements that, in their opinion, were similar in content. For this task, statements had to be distributed over more than one group. There was no restriction for the number of statements pertaining to one group.
4. Statistical analyses; These analyses were performed using Ariadne, a computer programme specifically designed to support concept mapping [37]. First, the arithmetic mean of the priorities that the participants assigned to each statement was calculated. This resulted in a list with ratings of statements. Then, a multidimensional scaling followed by hierarchical cluster analyses was used on the basis of a matrix of the categorizing results (i.e. how often two statements were placed together in the same category by participants). This resulted in a final set of clusters.
5. Interpretation of the concept map; This step consisted of determining the number of clusters and labelling the clusters, conducted by two independent researchers. Labelling was based on the content of the statements comprising the clusters.

The study has been designed as a qualitative study. The main criterion for the number of participants in qualitative studies is saturation. From this perspective about 10 to 20 persons are considered sufficient for the statement generating phase (step 2) in concept mapping [32], [38]. For the prioritization/categorization of statements (step 3) it is advised to have at least the same number, but groups of step 2 and 3 do not have to include the same persons [32].

Given the fact that our study is basically a qualitative study, we decided to start with a qualitative interpretation of our results. A strict qualitative approach of these data, however, will result in considerable loss of information. Therefore this was followed by quantitative testing, keeping in mind that the latter is hindered by low statistical power. For this reason we did not correct for multiple testing. Differences between mean priority ratings of stakeholders were tested with analysis of variance followed in case of a significant overall F test by multiple comparisons using Tukey method. All analyses were done with SPSS-18.

## Results

For the statement generation phase, 34 participants were invited to participate, of which 32 participants (94%) took part. For the prioritization and categorization phase, a total of 54 participants were invited to participate, of which 38 participants (70%) took part (Table 1). Participating employees and supervisors were working in healthcare (24%), finance (20%), education (16%) industry (12%) or other jobs (28%).

**Table 1:** Participants.

	Generation of statements	Categorizing and prioritizing
Participants	n (% men)	n (% men)
Employees	13 (46)	14 (43)
Supervisors	8 (38)	11 (45)
Occupational physicians	11 (36)	13 (54)
Total	32 (41)	38 (47)

**Figure 1:**
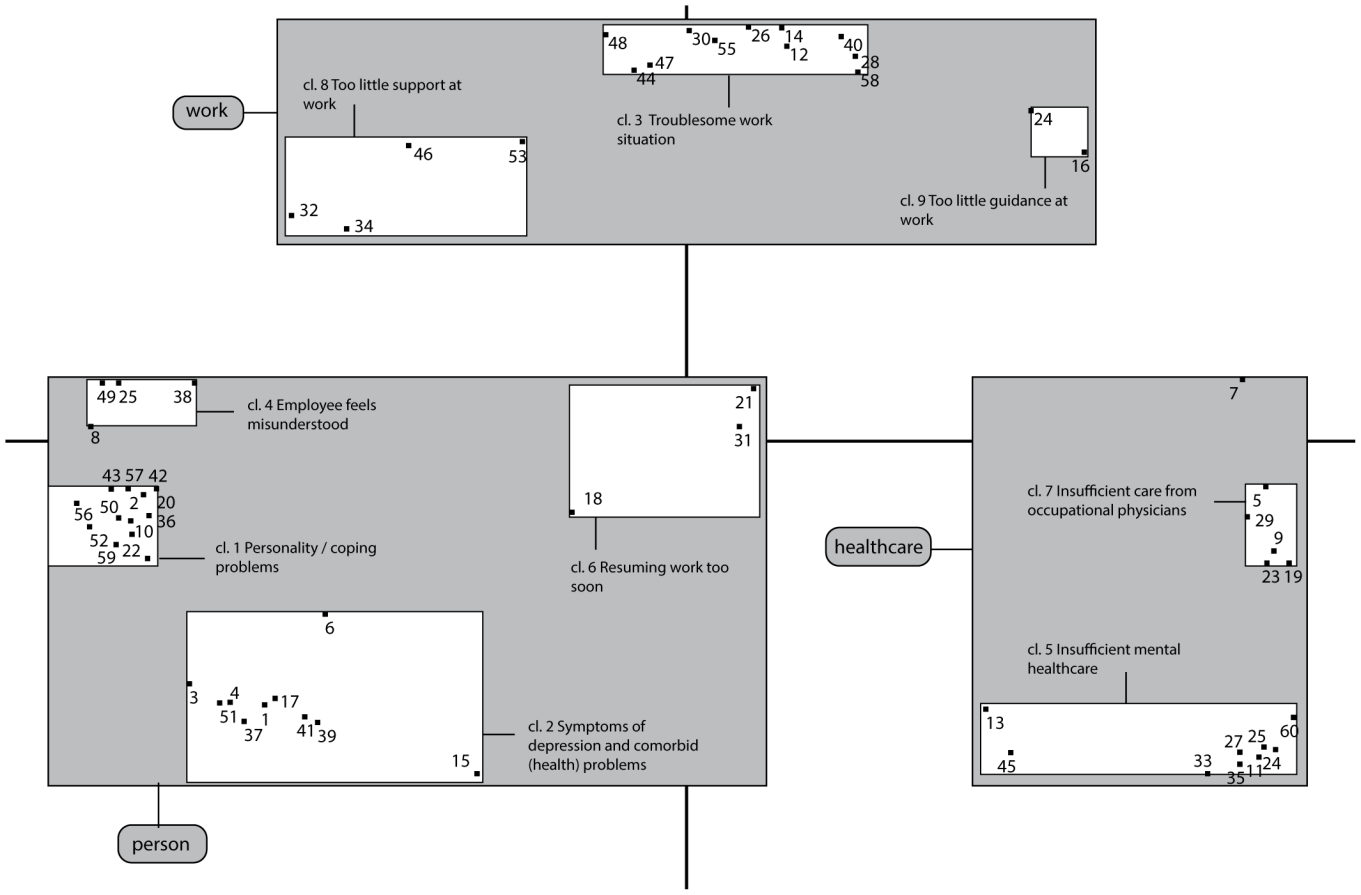
Concept map: Impeding factors for return-to-work (RTW) in employees with long-term sickness absence related to major depressive disorder (MDD); Statement numbers, clusters and meta-clusters.

In total, participants generated 373 statements. The number of statements varied between participants and was on average 11. After elimination of redundant, unrelated or unclear statements, a final set of 60 statements remained. These statements are presented as numbers in Figure 1 and written out in Table 2, 3 and 4.

**Table 2:** Meta cluster Person; Clusters and Statements.

Number	Clusters and Statements	Mean all participants
**Meta cluster A: Person**		3.1
**Cluster 1**	**Personality / coping problems**	3.2
42	Employee is hindered by factors such as being too demanding. too perfectionistic or having too little self-confidence	3.8
43	Employee is reluctant and avoids work resumption	3.6
36	Employee feels inferior. insecure and does not dare to assert themselves	3.5
50	Employee feels ashamed. a failure and is reluctant to return to work	3.5
57	Employee has difficulty facing problems and to reflect on his behavior. which hinders recovery	3.5
2	Employee does not accept his functioning is (has become) limited	3.2
20	There is lack of understanding and support from home (loneliness. relationship problems)	3.1
59	Employee externalizes the origin of his problems	3.1
10	Employee has difficulty indicating his needs	3.0
22	The employee has additional pressures at home (e.g. care for sick child. partner or parent)	3.0
52	Employees is not able to discuss his own functioning	2.8
56	Employees does not feel competent	2.8
**Cluster 2**	**Symptoms of depression and comorbid (health) problems**	3.2
1	Employee is still too depressed	4.1
3	Employee suffers from worrying. concentration or memory problems	3.7
4	Employee is too tired. has low energy	3.7
39	Besides depression employee has other psychiatric problems	3.5
51	Employee has had several periods of depression	3.4
41	Besides depression employee has also had problems with addiction	3.0
6	There are too many problems	2.8
17	Employee is also suffering from physical complaints	2.7
15	Employee suffers from side effects of medication	2.6
37	There are residual problems with a grieving process	2.5
**Cluster 4**	**Employee feels misunderstood**	3.0
25	Employee experiences too little protection and support	3.3
8	Employee does not feel understood	3.2
49	Employee feels to be put under pressure	2.8
38	Employee needs too much support	2.6
**Cluster 6**	**Resuming work too soon**	2.7
18	Employee resumes work too soon to succeed	3.4
31	Employee does not have the opportunity to recover mentally	2.7
21	Employee feels abandoned by employer and/or social legislation	2.0
		

**Table 3:** Meta cluster Work; Clusters and Statements.

Number	Clusters and Statements	Mean all participants
**Work**		2.9
**Cluster 3**	**Troublesome work situation**	3.1
26	Employer wants to get rid of employee	3.5
30	There is a (dormant) work dispute	3.4
47	Employee receives little support with his problems at work	3.3
48	Employee no longer fits into the organization	3.2
14	Supervisor demands too much from the employee	3.2
44	Employee is put under pressure at work	3.1
40	Employer does not offer suitable employment	3.1
58	Employer does not feel competent about the supervision process	2.9
55	Reorganizations at work	2.8
12	Employee and employer have discontinued their work relationship	2.8
28	Employer is not well informed enough (due to privacy) and is therefore unable to support the employee adequately	2.3
**Cluster 8**	**Too little support at work**	2.6
32	Employee experiences an unsafe work environment	3.1
46	Employee receives too little structure and guidance	2.9
34	Employee receives little support from colleagues	2.8
53	Employee is too old	1.8
**Cluster 9**	**Too little guidance at work**	2.5
24	Supervisor is not able to shape guidance sufficiently	3.2
16	Employer is hindered by legislation in the provision of appropriate work	1.7

**Table 4:** Meta cluster Healthcare; Clusters and Statements.

Number	Clusters and Statements	Mean all participants
**Health care**		2.9
**Cluster 5**	**Insufficient mental health care**	3.0
35	Treatment is insufficient or does not meet the need	3.2
33	Psychiatric advice not to resume work	3.2
54	The multi-professional team does not work together well enough	3.1
11	There is too little attention to work and return to work in mental health care	3.0
27	Health care starts too late. e.g. due to long waiting lists	2.9
45	Health care does not suit employees from ethnic minorities	2.9
60	There is insufficient collaboration in mental health care	2.7
13	Employee does not experience support from occupational physician and/or psychiatrist	2.7
**Cluster 7**	**Insufficient care from occupational physician**	2.6
9	There is insufficient collaboration between the employer and the occupational physician	3.2
19	Occupational physician does not intervene adequately	2.9
5	Reintegration is slowed down due to lack of support from supervisor and occupational physician	2.8
23	Occupational physician is not familiar with work environment	2.5
29	There is lack of support from social legislation	1.9
	**Not clustered statement**	3.0
7	No proper monitoring of the integration process	3.0

### Clusters

The hierarchal cluster analysis conducted by the Ariadne program resulted in two meaningful cluster solutions (Figure 1): a three- and a nine-cluster solution. We will refer to the clusters in the three-cluster solution as meta-clusters, described in Figure 1 as (A) Person, (B) Work and (C) Healthcare, and the clusters in the nine-cluster solution as clusters. The nine-cluster solution provides additional meaning to the three meta-clusters. The labeling of the clusters was based on the statements that comprised this cluster, with emphasis on the statements with the highest priority score. Numbering of the clusters is in order of their importance, which is based on the mean priority score of all statements within this cluster. Thus, cluster 1, “Personality/coping problems”, can be considered as the most important impeding cluster for RTW, and cluster 9, “Employer is unable to shape support sufficiently” can be considered as the least important cluster for impeding RTW. The score of the statements is based on the mean priority score over all participants.

Meta cluster A, “Person”, contains 29 statements grouped into four clusters, which all pertain to the individual employee (Table 2). Cluster 1, “Personality/coping problems”, comprises statements that refer to problems related to personality and coping that may impede the employee's RTW, such as perfectionism (st.42), difficulty to act assertively (st.36), avoidance/reluctance to RTW (st.43), and externalization of problems (st.59). Cluster 2, “Symptoms of depression and other comorbid (health) problems”, comprises of statements that refer to symptoms such as depressive feelings (st.1), cognitive problems (st.3) and low energy (st.4), and to comorbid health problems such as other psychiatric problems (st.39), problems with addiction (st.41), and physical complaints (st.17). Cluster 4, “Employee feels misunderstood”, refers to the employees' experiences that there is too less notion of the employee's opinion (st.8) and opportunities (st.25). Finally, cluster 6, “Resuming work too soon” refers to a too soon work resumption (st.18) and the lack of opportunity to recover mentally (st.31) as impeding RTW. Although cluster 4 and 6 were grouped in the meta-cluster Person, they were positioned in the quadrant nearby clusters that referred to the meta-cluster work environment, indicating that they pertain to the person but are also related to the work environment.

Meta-cluster B, “Work”, contains 17 statements grouped into three clusters, which all pertained to the work environment (Table 3). Cluster 3 “Troublesome work situation”, comprises statements that could lead to a wish to terminate employment, such as the employer who wants to get rid of the employee 

(st.26), the presence of a (dormant) work dispute (st.30), the employee who does no longer fit within the organisation (st.48), or a supervisor who is too demanding (st.14). Cluster 8, “Too little support at work”, refers to the employee experiencing an unsafe work environment (st.32), a lack of structure (st.46), and a lack of support from colleagues (st.34). Cluster 9, “Too little guidance at work”, refers to the inability of the employer to provide adequate guidance (st.24,16).

Meta-cluster C, “Healthcare”, contains 14 statements grouped into two clusters, which pertain to mental- and occupational healthcare (Table 4). Cluster 5, “Insufficient mental health care”, refers to insufficient treatment (st.35), negative psychiatric advice to resume work (st.33), insufficient attention for RTW in mental health care (st.11), and insufficient cooperation and collaboration between various healthcare professionals (st.54, 60). These statements may be considered as statements in which healthcare was unaware of, or inadequate in dealing with the demands of the work situation. Cluster 7, “Insufficient care from OP”, comprises statements that pertain to a lack of collaboration between the OP and employer (st.9) and a lack of support from the OP (st.19, 5).

### Differences between stakeholders

First we compared the percentage of most important statements (i.e. statements with a mean priority rating ≥ 3.5; these statements will further be referred to as ‘important statements’) pertaining to the meta-clusters and clusters (Table 5). Employees rated 9 statements as important statements, supervisors 13, and OP's 19. Employees put most emphasis on the meta-cluster “Person”: All their important statements pertained to this meta-cluster (100%). This was only the case for 60% of the supervisors and 52% of the OP's. Within the meta-cluster “Person”, employees and OP's considered more statements in cluster 2 (i.e., “Symptoms of depression and comorbid (health) problems”) important compared to supervisors. Supervisors considered more statements important that pertained to cluster 1, “Personality/coping problems”.

**Table 5:** Differences between stakeholder groups based on meta clusters, clusters and important statements (statements with a priority of ≥3.5 rated by at least one stakeholder group).

nr	Statement	Mean Empl.	Mean Superv.	Mean OP's**	p	F (2.35)	Tukey
	**Person**	3.2	3.1	3.1	0.65		
**Cl. 1**	**Personality / coping problems**	3.2	3.2	3.3	0.95	0.05	
42	Employee is hindered by factors such as being too demanding, too perfectionistic or having too little self-confidence	**4.1**	3.2	**4.2**	0.08	2.79	
36	Employee feels inferior, insecure and does not dare to assert themselves	**3.9**	3.2	**3.5**	0.35	1.09	
43	Employee is reluctant and avoids work resumption	**3.6**	**3.5**	**3.6**	0.96	0.04	
50	Employee feels ashamed, a failure and is reluctant to return to work	3.3	**3.9**	3.4	0.47	0.52	
2	Employee does not accept his functioning is (has become) limited	3.1	**3.5**	2.9	0.66	0.41	
20	There is lack of understanding and support from home (loneliness, relationship problems)	3.0	**3.5**	2.8	0.44	0.85	
57	Employee has difficulty facing problems and to reflect on his behavior, which hinders recovery	2.9	**3.8**	**3.9**	0.14	2.92	
**Cl. 2**	**Symptoms of depression and comorbid (health) problems**	3.3	3.0	3.2	0.39	0.97	
3	Employee suffers from worrying. concentration or memory problems	**4.4**	3.1	3.7	0.11	2.33	
4	Employee is too tired,. has low energy	**4.4**	3.2	**3.5**	0.07	2.9	
1	Employee is still too depressed	**4.3**	**4.1**	**3.9**	0.67	0.28	
6	There are too many problems	**3.7**	2.3	2.5	**0.01**	5.60	(1–2;1–3)
51	Employee has had several periods of depression	**3.6**	**3.6**	3.0	0.52	0.67	
39	Besides depression employee has other psychiatric problems	2.9	3.4	4.3	**0.05**	3.29	(1–3)
41	Besides depression employee also has problems with addiction	1.7	3.0	**4.2**	**<0.01**	10.92	(1–3)
**Cl. 4**	**Employee feels misunderstood**	2.9	3.0	3.1	0.65	0.44	
25	Employee experiences too little protection and support	3.1	2.7	**4.1**	**0.03**	4.04	(2–3)
**Cl. 6**	**Resuming work too soon**	3.2	2.8	2.2	**<0.01**	7.70	(1–3;2–3)
18	Employee resumes work too soon to succeed	**4.3**	**3.5**	2.5	**0.01**	5.39	(1–3)
	**Work**	2.9	2.9	2.9	0.95		
**Cl. 3**	**Troublesome work situation**	3.0	3.1	3.1	0.88	0.12	
26	Employer wants to get rid of employee	3.4	**3.5**	**3.5**	1.00	<0.01	
47	Employee receives little support with his problems at work	3.2	3.0	**3.8**	0.27	1.34	
44	Employee is put under pressure at work	2.9	2.9	**3.5**	0.32	1.17	
30	There is a (dormant) work dispute	2.6	**3.6**	**4.0**	**0.05**	3.25	(1–3)
**Cl. 8**	**Too little support at work**	2.9	2.3	2.7	0.27	1.37	
32	Employee experiences an unsafe work environment	3.2	2.3	**3.8**	**0.05**	3.33	(2–3)
**Cl. 9**	**Too little guidance at work**	2.4	2.6	2.5	0.74	0.30	
24	Supervisor is not able to shape guidance sufficiently	3.0	3.0	**3.6**	0.28	1.32	
	**Healthcare**	2.7	3.0	2.9	0.40		
**Cl. 5**	**Insufficient mental health care**	2.6	3.2	3.2	**0.02**	4.19	(1–2;1–3)
33	Psychiatric advice not to resume work	3.1	**3.9**	2.7	0.15	2.03	
11	There is too little attention to work and return to work in mental health care	2.8	2.8	**3.5**	0.29	1.29	
35	Treatment is insufficient or does not meet the need	2.7	**3.7**	3.3	0.14	2.12	
54	The multi-professional team does not work together well enough	2.6	**3.5**	3.2	0.34	1.10	
45	Health care does not suit employees from ethnic minorities	2.1	2.9	**3.7**	**0.01**	5.11	(1–3)
27	Health care starts too late. e.g. due to long waiting lists	2.2	3.0	**3.5**	**0.04**	3.41	(1–3)
**Cl. 7**	**Insufficient care from occupational physician**	2.9	2.7	2.3	0.29	1.29	

Statements are first ordered by meta-cluster, second by cluster, and third by highest mean score of employee stakeholder group.

OP = occupational physician.

Supervisors and OP's also rated some statements of the meta-clusters “Work” and “Healthcare” as important. OP's put more emphasis on the meta-cluster “Work”: Compared to supervisors, relatively more of their important statements pertained to this cluster (31 /15%). In addition, all statements ranked important by supervisors in the “Work” meta-cluster pertained to cluster 3, “Troublesome work situation”. Statements in this meta-cluster considered important by OP's also pertained to cluster 8 (“Too little support at work”) and cluster 9 (“Too little guidance at work”).

Although both supervisors and OP's considered three statements important from the meta-cluster “Healthcare” (i.e. cluster 5 “Insufficient mental healthcare”), the percentage of their important statements pertaining to this meta-cluster was higher for supervisors than for OP's (23% /16%). This is due to the larger total number of statements considered important by OP's.

Next we examined statistically differences in mean priority rating between stakeholders in meta-clusters, clusters and important statements (Table 5). No significant differences in mean priority rating were found between stakeholders at meta-cluster level. At cluster level, cluster 6 (“Resuming work too soon”) was considered more important by employees than by OP's (p<0.01). Cluster 5 (“Insufficient mental health care”) was more important for supervisors (p = 0.05) and OP's (p = 0.04) than for employees.

The higher importance of ‘too soon work resumption’ by employees compared to OP's is reflected by differences in the mean priority rating of statement 18 (“Employees resumes work too soon to succeed”; p = 0.01) and statement 6 (“There are too many problems”), which was considered more important to employees than to OP's (p = 0.03) and to supervisors (p = 0.01).This suggests that experienced problems might be more important for employees than for supervisors and OP's.

The higher importance of insufficient healthcare by OP's compared to employees is reflected by differences in mean priority rating on the following main statements: “Healthcare doesn’t suit employees from ethnic minorities” (st.45, p = 0.01) and “Healthcare starts too late” (st.27, p = 0.04). In addition, OP's considered other psychiatric problems (st.39, p = 0.04), problems with addiction (st.41, p<0.01) and a (dormant) work dispute (st.30, p = 0.05) more important than employees. Furthermore, they considered “Employees experiences too little protection and support” (st.25, p = 0.03) and “Employee experiences an unsafe work environment” (st.32, p = 0.04) as more important than supervisors. This suggests that although experienced problems of employees are also of main importance for OP's, they do not focus on experienced problems but rather on diagnosis, insufficient healthcare, and insufficient support from the work environment.

## Discussion

### General findings

This study examined what factors impede return-to-work (RTW) in employees with long-term sickness absence (LTSA) related to Major Depressive Disorder (MDD), from the perspectives of three key stakeholder groups; employees, supervisors and occupational physicians (OP's). In total, 60 statements were generated, grouped into nine clusters and three meta-clusters (Person, Work and Healthcare). Impeding clusters pertaining to the meta-cluster “Person” were: “Personality / coping problems”, “Symptoms of depression and comorbid (health) problems”, “Employee feels misunderstood”, and “Resuming work too soon”. Impeding clusters pertaining to the meta-cluster “Work” were: “Troublesome work situation”, “Too little support at work” and, “Too little guidance at work”. Impeding clusters pertaining to the meta-cluster “Healthcare” were: “Insufficient mental healthcare” and “Insufficient care from occupational physician”. The high number and wide range of impeding factors mentioned in the current study underline the multi-factorial nature and complexity of the RTW process after LTSA related to MDD [39].

Although stakeholders agreed on the importance of most clusters and statements, the present findings also indicate perceived differences in factors that contribute to delayed RTW. All statements regarded as important (≥3.5) by employees pertained to the meta-cluster Person (i.e., the clusters “Symptoms of depression” and “Personality/coping problems”). Of these, employees put most emphasis on the cluster “symptoms of depression”, in particular on too many problems (cl.2,st.6) and a too soon work resumption (cl.6, st.18). Although supervisors also considered most statements pertaining to this meta-cluster important, they put most emphasis on personality problems (cl.1). In addition, supervisors also considered statements related to the Work and Healthcare meta-cluster as important. OP's put also most emphasis on the meta-cluster Person in particular on co-morbidity (st.31, 41) and too little protection and support (st.25), followed by the Work meta-cluster, in particular a work dispute (st.30) and an unsafe work environment (st.32) and finally by insufficient mental healthcare due to too less attention for ethnic minorities (st.45) and too long waiting lists (st.27).

### Personality

Personality characteristics and coping style of the individual employee (i.e., cluster 1 “Personality/coping problems”) are regarded as an important impeding factor for RTW in sick-listed employees with MDD according to all three stakeholder groups. Personality traits such as little self-confidence (st.42, 50), feelings of inferiority (st.36, 56), and externalizing the origin of problems (st.59, 52) may be related to an avoidant and dependent personality, and may be indicative of Cluster C personality disorders on Axis II of the DSM-IV. Furthermore, these personality traits may affect coping strategies, thereby reinforcing the impediments on RTW [28]. These findings are consistent with previous studies in various health conditions, where low self-esteem, high neuroticism, low extraversion, perfectionism and external locus of control were found as predictors of long-term sickness absence [3], [19], [41], [42] and are risk factors for decreased work functioning [42], [43]. These impeding factors should be addressed by mental healthcare.

### Severity of depression

The present findings emphasize the importance of depressive symptoms and co-morbid health problems, impeding RTW. This is also supported by previous literature [4], [8], [18], [19] showing that symptom severity (e.g., concentration problems, low energy) and co-morbidity (e.g., anxiety or substance abuse) are important predictors of unsuccessful RTW. Care providers, however, should realize that symptom reduction will not lead to better RTW outcomes per se. A focus on symptoms can reinforce the illness identity and non-work identity of the employee, which in turn can have a negative effect on the RTW process [28]. In addition, the state of the art treatment of chronic diseases supporting RTW prescribes a focus on the ability to cope with symptoms related limitations within the (work) environment, alongside medical treatment, as for example proven by Individual Placement and Support (IPS), the most effective RTW method for severe mental health disorders [44]. Because recurrence of MDD is high [16], it is argued that MDD, especially persistent MDD, should be treated as a chronic disease [45]. In extent, work participation may have a positive effect on health [46], [47], [48], as it has a positive effect on perceived health for unemployed citizens receiving social security benefits [48] and for employees suffering from MDD [49]. Furthermore, work participation did not worsen the health status for employees suffering from severe mental health [50].

### Work relationship

Within the work environment, a troublesome relationship and too little support and guidance at work were found as important impeding factors for RTW in employees with long-term sickness absence related to MDD. Interestingly, these impeding factors do not consist of work characteristics that pertain to the amount and severity of tasks related to an increase in the incidence of MDD (i.e., high (psychological) work demands and low decision latitude [51], [52]) or on the absence of these factors that may support RTW in sick-listed employees with MDD (i.e., adjusting tasks and positive work experiences [30]). Rather, these impeding factors are more related to work characteristics that pertain to social support. In literature, low social support was found increasing the incidence of depression [51], [52], and previous studies showed also that a good relationship, such as goodwill and trust [53], safety feelings [54], and perceived support from the supervisor [40] are important factors for achieving successful RTW in other (mental) health conditions. Supervisors support and the relationship between employee and supervisor may not only be hindered by the employees' personality (e,g, the employee's lack of assertiveness or reluctance to RTW) but also by the supervisors' negative judgment about the employees' competence, as this competence may be negatively influenced by the symptoms of depression. Therefore, with delayed RTW, guidance should also focus on improving support and work relationships.

### Mental healthcare

From the perspective of supervisors and OP's, insufficient mental healthcare also contributed to a delayed RTW. They addressed the importance of having more attention for the work situation and RTW in mental healthcare (st.11, 33, 35), improved cooperation between different healthcare professionals (st.54), having more attention for ethnic minorities (st.45), and shortening of waiting lists (st.27). The importance of cooperation and attention for RTW in healthcare is also found in studies with other populations such as musculoskeletal conditions [55], and is one of the main elements of IPS for employees with severe mental health problems [50]. In addition, still about half of employees suffering from severe mental health disorders do not receive mental health at all, and if they did, many do not receive adequate treatment in line with minimum clinical guidelines [21]. Therefore, both the quantity and quality of mental healthcare can be improved, in particular for employees who are at risk for delayed RTW.

### Strengths and limitations

The strength of this study is the focus on the varying perspectives of different key stakeholders involved: employees, supervisors and OP's. Their personal experiences give insight into a wide range of factors that may impede the RTW process. To our knowledge, sick-listed employees with MDD have rarely been asked open-ended questions as to what they see as impediments for their RTW. In addition, the present study improves our knowledge by highlighting differences in key stakeholder perspectives.

Nevertheless, the current study also has some limitations. Although the number of participants is more than sufficient for the concept mapping procedure [32], [38], caution should be exercised for generalizing differences between stakeholder groups, as the number of participants within each stakeholder group is relatively small. Second, although personality/coping and depression symptoms were separated in two different clusters, with the current data, it cannot be concluded to what extent these personality traits/coping styles are related to the severity of MDD, or whether these traits exist independently of the MDD. Finally, when interpreting the current study findings, one should take into account that the legislative context in the Netherlands differs from other countries, which may have influenced study results [40]. Dutch legislation already has an active focus on RTW: after 6 weeks of sickness absence, the employee and supervisor are obligated to make a reintegration plan and OP's advice is required. In addition, in the Netherlands, the employer is legally obligated to pay at least 70% of the employee's salary during the first two years of sickness absence. Therefore, supervisors and OP's may have already used a relatively active approach for achieving RTW. The absence of this active approach may be a main impeding factor in other countries.

### Conclusion

In conclusion, the presence of depressive symptoms, personality/coping problems, a disturbed relationship at work and too little attention for the work environment in healthcare were perceived by stakeholders as the main impeding factors for RTW after long-term sickness absence (LTSA) related to major depressive disorder (MDD). Attention for these impeding factors in earlier phases of the RTW process may increase the opportunity to improve this RTW process, thereby preventing LTSA as well as unnecessary personal grief and loss of employees' social value.

## References

[1] Hensing G, Andersson L, Brage S (2006) Increase in sickness absence with psychiatric diagnosis in Norway: a general population-based epidemiologic study of age, gender and regional distribution. BMC Med 4: 19–28. 16923198 10.1186/1741-7015-4-19 PMC1601961

[2] Nielsen MB, Bültmann U, Madsen IE, Martin M, Christensen U, et al (2012) Health, work, and personal-related predictors of time to return to work among employees with mental health problems. Disabil Rehabil 34: 1311–1316. 22200251 10.3109/09638288.2011.641664

[3] Roelen CA, Norder G, Koopmans PC, van Rhenen W, van der Klink JJ, et al (2012) Employees sick-listed with mental disorders: who returns to work and when? J Occup Rehabil 22: 409–417. 22447276 10.1007/s10926-012-9363-3

[4] Vlasveld MC, van der Feltz-Cornelis CM, Bültmann U, Beekman AT, van Mechelen W, et al (2012) Predicting return to work in workers with all-cause sickness absence greater than 4 weeks: a prospective cohort study. J Occup Rehabil 22: 118–126. 21842133 10.1007/s10926-011-9326-0 PMC3274679

[5] Cornelius LR, van der Klink JJ, Groothoff JW, Brouwer S (2011) Prognostic factors of long term disability due to mental disorders: a systematic review. J Occup Rehabil 21: 259–274. 21057974 10.1007/s10926-010-9261-5 PMC3098343

[6] Nieuwenhuijsen K, Verbeek JH, de Boer AG, Blonk RW, van Dijk FJ (2006) Predicting the duration of sickness absence for patients with common mental disorders in occupational health care. Scand J Work Environ Health 32: 67–74. 16539174 10.5271/sjweh.978

[7] Lépine JP, Briley M (2011) The increasing burden of depression. Neuropsychiatr Dis Treat (Suppl 1): 3–7. 10.2147/NDT.S19617 PMC3131101 21750622

[8] Bültmann U, Christensen KB, Burr H, Lund T, Rugulies R (2008) Severe depressive symptoms as predictor of disability pension: a 10-year follow-up study in Denmark. Eur J Public Health 18: 232–234. 18202084 10.1093/eurpub/ckm132

[9] Goetzel RZ, Ozminkowski RJ, Sederer LI, Mark TL (2002) The business case for quality mental health services: why employers should care about the mental health and well-being of their employees. J Occup Environ Med 44: 320–330. 11977418 10.1097/00043764-200204000-00012

[10] Glozier N (2002) Mental ill health and fitness for work. Occup Environ Med 59: 714–720. 12356935 10.1136/oem.59.10.714 PMC1740218

[11] O'Neil A, Sanderson K, Oldenburg B (2010) Depression as a predictor of work resumption following myocardial infarction (MI): a review of recent research evidence. Health Qual Life Outcomes 8: 95. 20815937 10.1186/1477-7525-8-95 PMC2944344

[12] Hansson E, Hansson T, Jonsson R (2006) Predictors for work ability and disability in men and women with low-back or neck problems. Eur Spine J 15: 780–793. 15937677 10.1007/s00586-004-0863-5 PMC3489465

[13] Lerner D, Henke RM (2008) What does research tell us about depression, job performance, and work productivity? J Occup Environ Med 50: 401–410. 18404013 10.1097/JOM.0b013e31816bae50

[14] Wang PS, Beck AL, Berglund P, McKenas DK, Pronk NP, et al (2004) Effects of major depression on moment-in-time work performance. Am J Psychiatry 161: 1885–1891. 15465987 10.1176/ajp.161.10.1885

[15] Koopmans PC, Roelen CA, Groothoff JW (2008) Sickness absence due to depressive symptoms. Int Arch Occup Environ Health 81: 711–719. 17849142 10.1007/s00420-007-0243-7 PMC2254471

[16] Hardeveld F, Spijker J, De Graaf R, Nolen WA, Beekman ATF (2010) Prevalence and predictors of recurrence of major depressive disorder in the adult population. Acta Psychiatr Scand 122: 184–191. 20003092 10.1111/j.1600-0447.2009.01519.x

[17] Spijker J, Graaf R, Bijl RV, Beekman AT, Ormel J, et al (2004) Functional disability and depression in the general population. Results from the Netherlands Mental Health Survey and Incidence Study (NEMESIS). Acta Psychiatr Scand 110: 208–214. 15283741 10.1111/j.1600-0447.2004.00335.x

[18] Hees HL, Koeter MW, Schene AH (2012) Predictors of long-term return to work and symptom remission in sick-listed patients with major depression. J Clin Psychiatry 73: e1048–1055. 22967781 10.4088/JCP.12m07699

[19] Lagerveld SE, Bültmann U, Franche RL, van Dijk FJ, Vlasveld MC, et al (2010) Factors associated with work participation and work functioning in depressed workers: a systematic review. J Occup Rehabil 20: 275–292. 20091105 10.1007/s10926-009-9224-x PMC2923705

[20] van der Werff E, Verboom CE, Penninx BW, Nolen WA, Ormel J (2010) Explaining heterogeneity in disability associated with current major depressive disorder: effects of illness characteristics and comorbid mental disorders. J Affect Disord 127: 203–210. 20594596 10.1016/j.jad.2010.05.024

[21] OECD Sick on the Job? Myths and Realities about Mental Health and Work, Mental Health and Work, OECD Publishing, 2012, Available: http://dx.doi.org/10.1787/9789264124523-en.

[22] Elinson L, Houck P, Marcus SC, Pincus HA (2004) Depression and the ability to work. Psychiatr Serv 55: 29–34. 14699197 10.1176/appi.ps.55.1.29

[23] Nieuwenhuijsen K, Bultmann U, Neumeyer-Gromen A., Verhoeven AC, Verbeek JH, et al.. (2008) Interventions to improve occupational health in depressed people. Cochrane. Database. Syst. Rev. (2), CD006237. 10.1002/14651858.CD006237.pub2 18425942

[24] International Classification of Functioning, Disability, and Health (2001) ICF full version. Geneva, Switzerland: World Health Organization.

[25] Waddell G, Burton AK (2005) Concepts of rehabilitation for the management of low back pain. Best Pract Res Clin Rheumatol 19: 655–670. 15949782 10.1016/j.berh.2005.03.008

[26] Blank L, Peters J, Pickvance S, Wilford J, Macdonald E (2008) A systematic review of the factors which predict return to work for people suffering episodes of poor mental health. J Occup Rehabil 18: 27–34. 18213510 10.1007/s10926-008-9121-8

[27] Nielsen MB, Madsen IE, Bültmann U, Christensen U, Diderichsen F, et al (2011) Predictors of return to work in employees sick-listed with mental health problems: findings from a longitudinal study. Eur J Public Health 21: 806–811. 21126986 10.1093/eurpub/ckq171

[28] Andersen MF, Nielsen KM, Brinkmann S (2012) Meta-synthesis of qualitative research on return to work among employees with common mental disorders. Scand J Work Environ Health 38: 93–104. 22025244 10.5271/sjweh.3257

[29] Dekkers-Sánchez PM, Wind H, Sluiter JK, Hw Frings-Dresen MH (2011) What promotes sustained return to work of employees on long-term sick leave? Perspectives of vocational rehabilitation professionals. Scand J Work Environ Health 37: 481–493. 21667007 10.5271/sjweh.3173

[30] de Vries G, Koeter MW, Nabitz U, Hees HL, Schene AH (2012) Return to work after sick leave due to depression; a conceptual analysis based on perspectives of patients, supervisors and occupational physicians. J Affect Disord 136: 1017–1026. 21774988 10.1016/j.jad.2011.06.035

[31] Hees HL, Nieuwenhuijsen K, Koeter MW, Bültman U, Schene AH (2012) Towards a new definition of return-to-work outcomes in common mental disorders from a multi-stakeholder perspective. Plos One 7: 1–7. 10.1371/journal.pone.0039947 PMC3386986 22768180

[32] Kane M, Trochim,WMK (2007) Concept mapping for planning and evaluation. London: Sage Publications Inc.

[33] Rosas SR, Kane M (2012) Quality and rigor of the concept mapping methodology: a pooled study analysis. Eval Program Plann 35: 236–245. 22221889 10.1016/j.evalprogplan.2011.10.003

[34] Williams RM, Westmorland M (2002) Perspectives on workplace disability management: a review of the literature. Work 19: 87–93. 12454354

[35] Integrated report of Stress Impact: On the impact of changing social structures on stress and quality of life: Individual and social perspectives (2006) The Stress Impact Consortium Work Package 8 Integrated report. Available: www.Surrey.ac.uk/Psychology/stressimpact.

[36] Nabitz U, van Den Brink W, Jansen P (2005) Using concept mapping to design an indicator framework for addiction treatment centres. Int J Qual Health Care 17: 193–201. 15831547 10.1093/intqhc/mzi037

[37] Severens P (1995) Handbook Concept mapping. Amsterdam: Talcott. National Centre of Metal Health.

[38] Southern DM, Batterham RW, Appleby NJ, Young D, Dunt D, et al (1998) The concept mapping method: An alternative to focus group inquiry in general practice. Aust Fam Physician 28: S35–S40. 9988927

[39] Franche RL, Krause N (2002) Readiness for return to work following injury or illness: conceptualizing the interpersonal impact of health care, workplace, and insurance factors. J Occup Rehabil 12: 233–256. 12389476 10.1023/a:1020270407044

[40] D'Amato A, Zijlstra F (2010) Toward a climate for work resumption: the nonmedical determinants of return to work. J Occup Environ Med 52: 67–80. 20042881 10.1097/JOM.0b013e3181c75291

[41] Noordik E, Nieuwenhuijsen K, Varekamp I, van der Klink JJ, van Dijk FJ (2011) Exploring the return-to-work process for workers partially returned to work and partially on long-term sick leave due to common mental disorders: a qualitative study. Disabil Rehabil 33: 1625–1635. 21171843 10.3109/09638288.2010.541547

[42] Vlasveld MC, van der Feltz-Cornelis CM, Anema JR, van Mechelen W, Beekman AT, et al. (2013) The associations between personality characteristics and absenteeism: A cross-sectional study in workers with and without depressive and anxiety disorders. J Occup Rehabil7 23: :309– 31. 10.1007/s10926-012-9406-9 23179746

[43] Michon HW, ten Have M, Kroon H, van Weeghel J, de Graaf R, et al (2008) Mental disorders and personality traits as determinants of impaired work functioning. Psychol Med 38: 1627–1637. 18205968 10.1017/S0033291707002449

[44] Latimer EA, Lecomte T, Becker DR, Drake RE, Duclos I, et al (2006) Generalisability of the individual placement and support model of supported employment: results of a Canadian randomised controlled trial. Br J Psychiatry 189: 65–73. 16816308 10.1192/bjp.bp.105.012641

[45] Andrews G (2001) Should depression be managed as a chronic disease? BMJ 322: 419–421. 11179166 10.1136/bmj.322.7283.419 PMC1119639

[46] Lagerveld SE, Blonk RW, Brenninkmeijer V, Wijngaards-de Meij L, Schaufeli WB (2012) Work-focused treatment of common mental disorders and return to work: a comparative outcome study. J Occup Health Psychol. 17: 220–34. 10.1037/a0027049 22308965

[47] Huijs JJM, Koppes LJ, Taris TW, Blonk RWB (2012) Differences in Predictors of Return to Work Among Long-Term Sick-Listed Employees with Different Self-Reported Reasons for Sick Leave. J Occup Rehabil 22: 301–311. 22302668 10.1007/s10926-011-9351-z

[48] Schuring M, Mackenbach J, Voorham T, Burdorf AJ (2011) The effect of re-employment on perceived health. Community Health 65: 639–644. 10.1136/jech.2009.103838 20805192

[49] Hees HL, de Vries G, Koeter MW, Schene AH (2013) Adjuvant occupational therapy improves long-term depression recovery and return-to-work in good health in sick-listed employees with major depression: results of a randomised controlled trial. Occup Environ Med 70: 252–260. 23117218 10.1136/oemed-2012-100789

[50] Bond GR, Drake RE, Becker DR (2012) Generalizability of the Individual Placement and Support (IPS) model of supported employment outside the US. World Psychiatry 11: 32–39. 22295007 10.1016/j.wpsyc.2012.01.005 PMC3266767

[51] Bonde JP (2008) Psychosocial factors at work and risk of depression: a systematic review of the epidemiological evidence. Occup Environ Med 65: 38–445. 18417557 10.1136/oem.2007.038430

[52] Netterstrøm B, Conrad N, Bech P, Fink P, Olsen O, et al (2008) The relation between work-related psychosocial factors and the development of depression. Epidemiol Rev 30: 118–132. 18587142 10.1093/epirev/mxn004

[53] MacEachen E, Clarke J, Franche RL, Irvin E (2006) Workplace-based Return to Work Literature Review Group Systematic review of the qualitative literature on return to work after injury. Scand J Work Environ Health 32: 257–269. 16932823

[54] Franche RL, Baril R, Shaw W, Nicholas M, Loisel P (2005) Workplace-based return-to-work interventions: optimizing the role of stakeholders in implementation and research. J Occup Rehabil 15: 525–542. 16254753 10.1007/s10926-005-8032-1

[55] Briand C, Durand MJ, St-Arnaud L, Corbière M (2008) How well do return-to-work interventions for musculoskeletal conditions address the multicausality of work disability? J Occup Rehabil. 18: 207–217. 18392925 10.1007/s10926-008-9128-1

